# Feasibility and safety of specimen extraction *via* an enlarged (U-Plus) skin bridge loop ileostomy: a single-center retrospective comparative study

**DOI:** 10.3389/fonc.2023.1273499

**Published:** 2023-11-08

**Authors:** Shang Xiang, Shujuan Huang, Hui Ye, Wei Lu, Xiangsheng Zeng

**Affiliations:** ^1^ Department of Colorectal and Anal Surgery, Jingzhou Hospital Affiliated to Yangtze University, Jingzhou, China; ^2^ Department of Respiratory and Critical Care Medicine, Jingzhou Hospital Affiliated to Yangtze University, Jingzhou, China

**Keywords:** skin bridge loop ileostomy, specimen extraction, low rectal cancer, parastomal hernia, stoma quality of life

## Abstract

**Objective:**

To investigate the feasibility and safety of specimen extraction *via* an enlarged (U-Plus) skin bridge loop ileostomy.

**Methods:**

A retrospective analysis of 95 patients with rectal cancer who underwent laparoscopic low anterior rectal resection and skin bridge loop ileostomy between August 2018 and August 2022, including 44 patients with specimen extraction *via* an enlarged (U-Plus) skin bridge loop ileostomy (experimental group) and 51 patients with specimen extraction *via* an abdominal incision (control group). Following the application of propensity score matching (PSM), 34 pairs of data were successfully matched. Subsequently, a comparative analysis was conducted on the clinical data of the two groups.

**Results:**

The experimental group exhibited significantly better outcomes than the control group in various aspects. Specifically, the experimental group had lower values for average operative time (*P* < 0.001), estimated blood loss (*P* < 0.001), median length of visible incision after surgery (*P* < 0.001), median VAS pain score on the first day after surgery (*P* = 0.015), and average postoperative hospitalization (*P* = 0.001). There was no statistical significance observed in the incidence of stoma-related complications in both groups (*P* > 0.05). Within each group, the stoma-QOL scores before stoma closure surgery were significantly higher than those at one month and two months after the surgery, with statistical significance (*P* < 0.05).

**Conclusion:**

Specimen extraction *via* a U-Plus skin bridge loop ileostomy is a safe and feasible method that shortens operation time and postoperative visual incision length, decreases estimated blood loss, and reduces patient postoperative pain compared with specimen extraction *via* an abdominal incision.

## 1 Introduction

The routine procedure for the treatment of low rectal cancer internationally is still laparoscopic low anterior resection (LAR), but anastomotic leak (AL) is one of the most serious complications of this procedure (1). And it is now generally accepted that protected ileostomy reduces the incidence of clinically relevant AL and the risk of unplanned reoperation without increasing mortality (2, 3). Ileostomy can also lead to complications such as skin inflammation around the stoma, necrosis, leakage, retraction, stenosis, prolapse, and parastomal hernia, which can reduce the quality of life of the patient if they occur (4, 5). Experts have proposed an approach that involves the one-stitch method of protective loop ileostomy, aimed at effectively reducing the surgical duration required for stoma creation while minimizing complications such as skin-mucosa separation, fecal dermatitis, and stoma retraction (6). And some experts have proposed a skin bridge loop ileostomy, which eliminates the need for a stoma rod compared to a traditional loop ileostomy, eliminates the postoperative operation associated with removing the stoma rod, and also reduces postoperative stoma-related complications and the frequency of stoma bag changes, increasing patient satisfaction (7–9).

During laparoscopic low anterior resection for rectal cancer, removing the specimen from the body is a crucial step. Currently, the most prevalent approach involves making an auxiliary incision in the abdomen to facilitate the specimen’s extraction. Additionally, there is literature supporting the use of Natural Orifice Specimen Extraction Surgery (NOSES) or specimen extraction *via* the ileostomy site to enhance the surgical outcome (10–12). In some cases, performing NOSES in patients with larger tumors or thickened intestinal walls and mesentery can be challenging or unsuccessful. Extracting the specimen through an ostomy also requires enlarging the abdominal wall incision at the ostomy site. Currently, there need to be more international studies investigating the feasibility and safety of enlarging the ostomy site for specimen extraction. Furthermore, no reports regarding specimen extraction *via* a skin bridge loop ileostomy have been found. This study aims to compare the advantages and disadvantages of specimen extraction through an enlarged (U-Plus) skin bridge loop ileostomy and a conventional auxiliary incision in the abdominal region. The aim is to explore the feasibility and safety of enlarging the abdominal wall incision at the loop ileostomy site for specimen extraction in patients with larger tumors or thickened intestinal walls and mesentery. This research provides a basis for selecting the approach and method of specimen extraction after laparoscopic LAR.

## 2 Methods

### 2.1 General information

**Figure 1:**
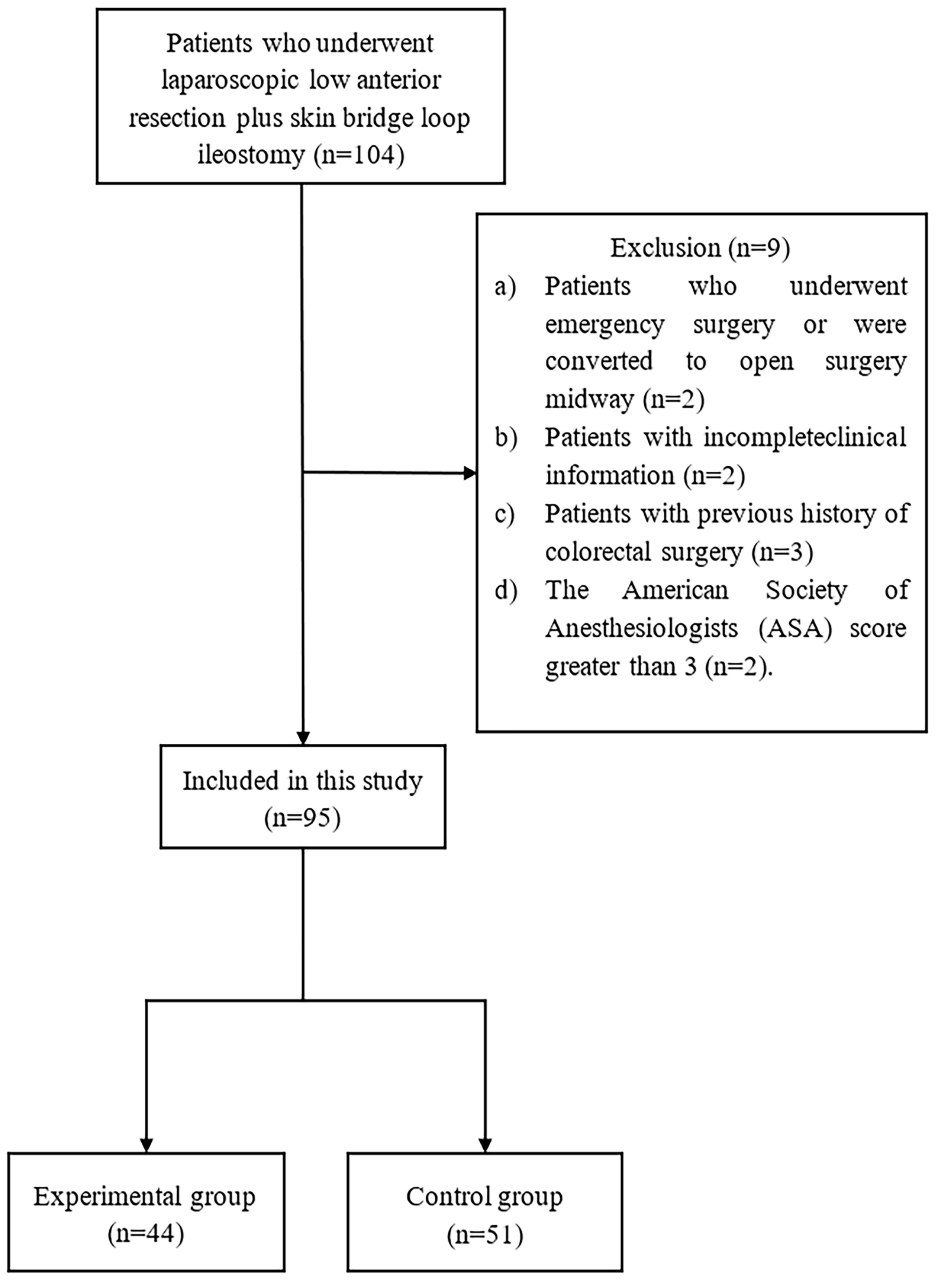
Flowchart of the screening process for included studies.

This study conducted a retrospective analysis of clinical data from 95 patients diagnosed with rectal cancer and undergoing laparoscopic low anterior resection and skin bridge loop ileostomy at Jingzhou Hospital Affiliated to Yangtze University between May 2018 and August 2022 (**Figure 1**). The study included 54 male and 41 female patients with a median age of 63 (52-74) years. The patients were divided into two groups based on the different methods of specimen extraction: the experimental group, comprising 44 cases of specimen extraction *via* a U-Plus skin loop ileostomy, and the control group, including 51 cases of specimen extraction through an abdominal incision. This study was approved by the Ethics Review Board of Jingzhou Hospital Affiliated to Yangtze University (2022-113-01) and was conducted by the ethical standards of the Helsinki Declaration.

### 2.2 Inclusion and exclusion criteria

#### 2.2.1 Inclusion criteria

(1) Preoperative diagnosis of primary rectal cancer; (2) Tumor located within 5cm from the anal verge; (3) Staging according to the 8th edition of the American Joint Committee on Cancer (AJCC) as stage I to III; (4) Patients with locally advanced disease (stage ≥ T3) received preoperative neoadjuvant therapy.

#### 2.2.2 Exclusion criteria

(1) Emergency surgery or conversion to open surgery during the procedure; (2) Incomplete medical records; (3) History of previous colorectal surgery; (4) The American Society of Anesthesiologists (ASA) score greater than 3.

### 2.3 Surgical procedure

#### 2.3.1 Control group

**Figure 2:**
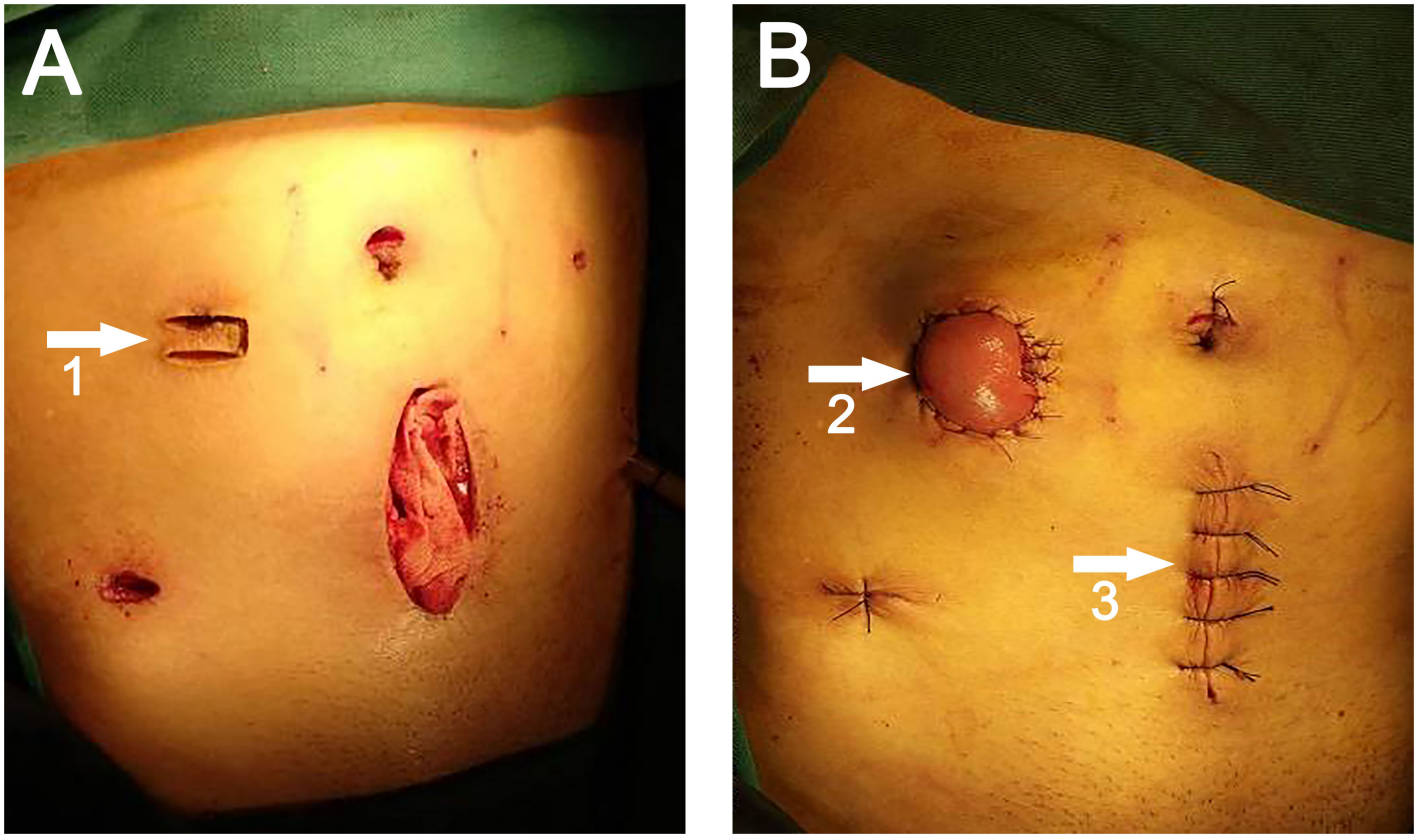
**(A)** White arrow 1 points to the U-shaped incision. **(B)** White arrow 2 points to the site of the ileostomy, white arrow 3 points to the site of specimen extraction.

All patients underwent preoperative stoma site marking by specialized ostomy nurses and surgeons. Before the commencement of laparoscopic surgery, the primary surgeon’s ancillary trocar placement was determined at the pre-marked stoma site on the right side of the mid-abdomen. Following the completion of the conventional laparoscopic LAR, a midline incision was made in the lower abdomen, with the length determined by the tumor size (6-10 cm). An incision protector was placed, and the rectal specimen was extracted using oval forceps *via* the incision protector. The sigmoid mesocolon should be trimmed with laparoscopic guidance to facilitate specimen extraction. Once the specimen was extracted, the incision protector was covered with a rubber glove to seal the abdominal cavity, carbon dioxide pneumoperitoneum was reestablished, and an end-to-end anastomosis with circular stapler was performed under laparoscopic guidance. After the anastomosis was completed, the assistant lifted the distal ileum (about 20-30 cm from the ileocecal region) using an atraumatic clamp under direct laparoscopic visualization. The surgeon then created a transverse rectangular skin flap with a pedicle (length 2.5-3.0 cm, width 1.0-1.5 cm, pedicle on the lateral side, U-shaped) using a scalpel along the right auxiliary trocar hole (**Figure 2A**). The subcutaneous adipose tissue was excised, and the external oblique aponeurosis was incised longitudinally to the peritoneum to enter the abdominal cavity. The prepared bowel segment was then pulled out through this incision, elevating 2-3 cm above the skin, with the proximal end positioned superiorly and the distal end inferiorly. The bowel, mesentery, peritoneum, and external oblique aponeurosis were intermittently sutured together. The pre-prepared rectangular skin flap was passed through the mesentery below the bowel and sutured to the opposite skin with 2-3 stitches of 3-0 Vicryl sutures, forming a skin bridge. The bowel wall and the surrounding skin of the stoma were also intermittently sutured with the same suture material. The stoma was then opened along the longitudinal axis of the bowel wall. Routine suturing of lower abdominal (**Figure 2B**) and cavity wounds.

#### 2.3.2 Experimental group

**Figure 3:**
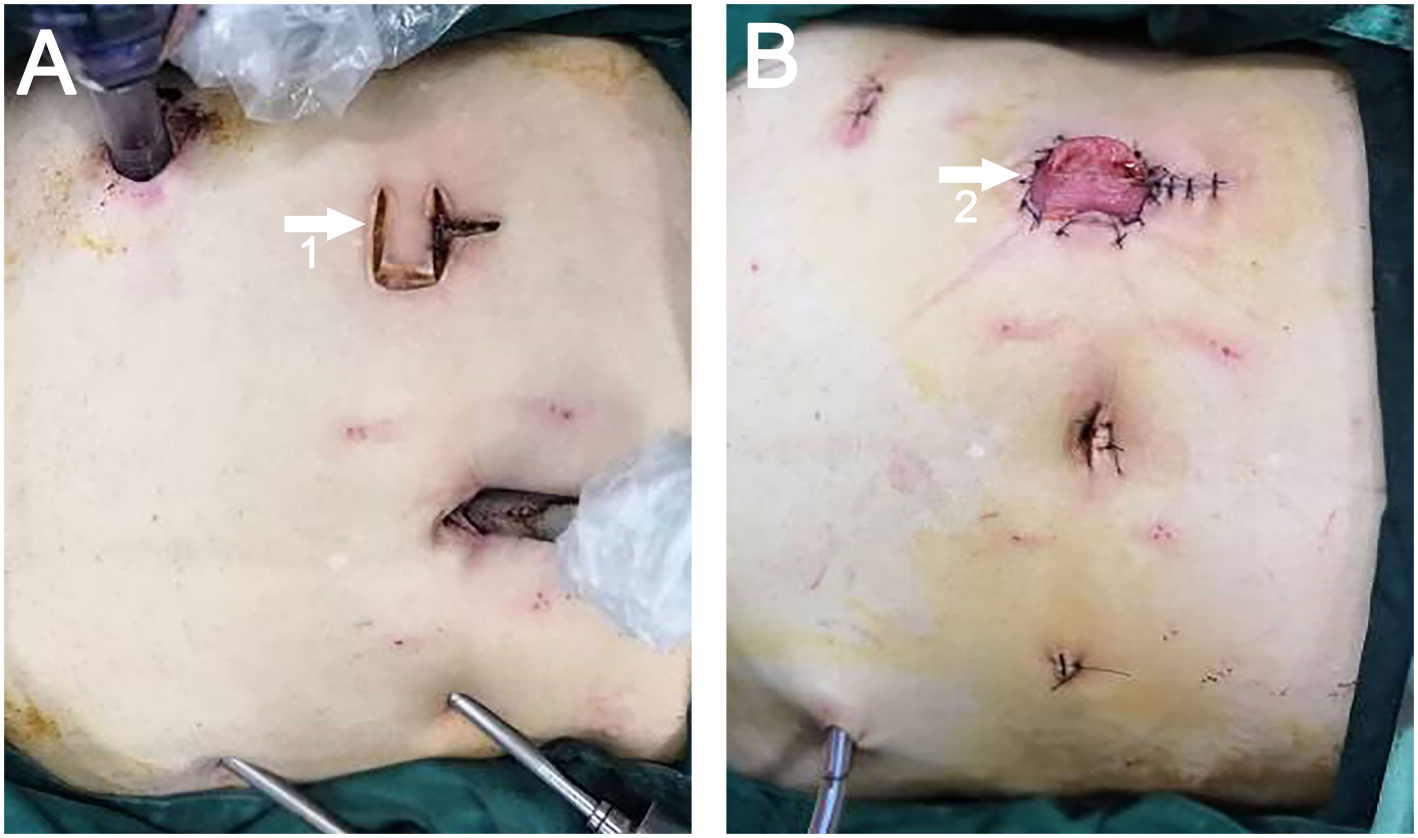
**(A)** White arrow 1 points to the U-Plus incision. **(B)** White arrow 2 points to the site of ileostomy and specimen extraction.

Adhering to the standard technique for laparoscopic LAR, a transverse U-shaped incision was executed in the preselected ostomy site on the right side of the abdomen, mirroring the approach employed for the control group. In the direction proximal to the patient’s head, the U-shaped incision extended longitudinally upward from the center (the U-plus incision) (**Figure 3A**). The magnitude of this extension was predicated on the diameter of the intestinal tract or the tumor’s dimensions, necessitating corresponding attachments in the subcutaneous tissue, muscle, and peritoneum. Subsequently, an incision protector was placed, enabling specimen extraction from this location (**Figure 3B**). The protocol for specimen extraction, intestinal anastomosis, and stoma formation was implemented in alignment with the techniques prescribed for the control group.

### 2.4 Follow-up time and observation indicators

The follow-up period was extended from patients’ discharge to stoma closure. The collected data encompassed three main components. Firstly, patients’ demographic information was gathered, including gender, age, Body mass index (BMI), preoperative levels of serum carcinoembryonic antigen (CEA) and carbohydrate antigen (CA) 199, pathological tumor-node-metastasis (pTNM) stage, ASA score, history of neoadjuvant chemotherapy, tumor distance from the anal verge, history of diabetes, and hypertension. Secondly, intraoperative and postoperative relevant indicators were recorded, such as operation time, estimated blood loss, maximum tumor diameter, postoperative visual incision length, rates of positive distal resection margin (DRM) and circumferential radial margin (CRM), time to first flatus, visual analogue scale (VAS) score on the first postoperative day, and postoperative hospitalization. Lastly, stoma-related parameters were documented, including stoma-related complications (stoma retraction, stoma stenosis, stoma infection, stoma necrosis, stoma edema, skin inflammation around the stoma, and parastomal hernia), peristomal dermatitis evaluation score (DET score) before stoma closure surgery, and stoma quality of life (Stoma-QOL) scores at one month, two months postoperatively and before stoma closure operation to assess stoma-related quality of life.

Pain scores were evaluated using the VAS score (13), with 0 indicating no pain and 10 indicating extreme pain. The Stoma Skin Tool (14) was utilized as a standardized measurement tool to assess the degree and severity of peristomal skin changes, including discoloration (D), erosion (E), and tissue overgrowth (T) (DET). Each domain was scored on a scale of 0 to 5, and the total DET score ranged from 0 to 15, with higher scores indicating more severe dermatitis. The Stoma-QOL score (15) assessed the quality of life in stoma patients across four dimensions: bowel-related problems, daily functioning, psychological impact, and social relationships. The total score ranged from 20 to 80, with higher scores indicating a better quality of life for patients.

### 2.5 Statistical analysis

To mitigate the impact of potential confounding variables on the outcomes, PSM was utilized for the variables within the patient data incorporated in the study. The statistical analysis was executed using IBM SPSS Statistics for Windows version 26.0 (IBM Corp, Armonk, NY, United States), employing a 1:1 nearest neighbor matching approach and establishing a caliper value of 0.02. The factors considered for matching encompassed: age, BMI, preoperative CEA and CA199 levels, pTNM stage, ASA score, history of neoadjuvant chemoradiotherapy, distance of tumor to anal verge, and medical histories of diabetes and hypertension. Statistical analysis was performed after PSM between the two groups. Normally distributed continuous variables were represented as mean ± SD and analyzed using the Student’s *t*-test. Non-normally distributed continuous variables were presented as the median and range [M (*Q_L_*, *Q_U_*)] and analyzed using the Mann-Whitney U test. Categorical variables were expressed as numbers and percentages and analyzed using the chi-square test, chi-square test with continuity correction, or Fisher’s exact test. A two-sided *P*-value < 0.05 was considered statistically significant for detecting differences.

## 3 Results

### 3.1 Patient characteristics before and after PSM

**Table 1** presents the basic patient information before the implementation of PSM. After balancing the parameters through PSM, 34 data pairs were successfully matched. Post-PSM, no statistically significant differences (*P* > 0.05) were found between the two patient groups in terms of gender, age, BMI, preoperative CEA, CA199, pTNM stage, ASA score, neoadjuvant radiochemotherapy, tumor distance from the anal margin, history of diabetes, and history of hypertension (**Table 2**).

**Table 1:** Patient characteristics before PSM.

Characteristic	Experimental group (n=44)	Control group (n=51)	*P*
Gender			0.675
Male	24 (54.4%)	30 (58.8%)	
Female	20 (45.5%)	21 (41.2%)	
Age (years)	62.1 ± 5.2	62.8 ± 5.3	0.498
BMI (kg/m^2^)	24.0 ± 1.1	24 ± 1.2	0.403
CEA (ng/ml)	8.6 ± 3.2	8.3 ± 3.1	0.684
CA199 (U/ml)	24.4 ± 5.1	22.4 ± 5.3	0.071
pTNM stage			0.893
I	6 (13.6%)	7 (13.7%)	
II	21 (42.7%)	22 (43.1%)	
III	17 (38.6%)	22 (43.1%)	
ASA score			0.665
1	4 (9.1%)	6 (11.8%)	
2	29 (65.9%)	29 (56.9%)	
3	11 (25.0%)	16 (31.4%)	
Distance of tumor to anal verge (cm)	4.5 (3.0-6.5)	4.0 (3.0-6.0)	0.930
Diabetes mellitus	7 (15.9%)	8 (15.7%)	0.976
Hypertension	12 (27.3%)	17 (33.3%)	0.522
Neoadjuvant therapy	22 (50.0%)	24 (47.1%)	0.775

BMI, Body mass index; CEA, Carcinoembryonic antigen; CA199, Carbohydrate antigen 199; pTNM, Pathological tumor-node-metastasis; ASA, American Society of Anesthesiologists.

**Table 2:** Patient characteristics after PSM.

Characteristic	Experimental group (n=34)	Control group (n=34)	*P*
Gender			0.083
Male	17 (50.0%)	24 (70.6%)	
Female	17 (50.0%)	10 (29.4%)	
Age (years)	62.0 ± 5.8	62.4 ± 5.7	0.794
BMI (kg/m^2^)	24.0 ± 1.0	24.1 ± 1.4	0.599
CEA (ng/ml)	8.8 ± 3.1	8.4 ± 3.3	0.704
CA199 (U/ml)	23.0 ± 4.5	23.6 ± 5.0	0.602
pTNM stage			0.967
I	4 (11.8%)	4 (11.8%)	
II	16 (47.1%)	15 (44.1%)	
III	14 (41.2%)	15 (44.1%)	
ASA score			
1	3 (8.8%)	3 (8.8%)	0.855
2	21 (61.8%)	23 (67.6%)	
3	10 (29.4%)	8 (23.5%)	
Distance of tumor to anal verge (cm)	4.3 ± 0.8	4.4 ± 0.7	0.586
Diabetes mellitus	3 (8.8%)	7 (20.7%)	0.171
Hypertension	9 (26.5%)	9 (26.5%)	1.000
Neoadjuvant therapy	15 (44.1%)	16 (47.1%)	0.808

BMI, Body mass index; CEA, Carcinoembryonic antigen; CA199, Carbohydrate antigen 199; pTNM, Pathological tumor-node-metastasis; ASA, American Society of Anesthesiologists.

### 3.2 Intraoperative and postoperative relevant indicators after PSM

All surgeries were successfully performed by experienced surgeons. Intraoperative and postoperative results after PSM are summarized in **Table 3**. The average operative time in the experimental group was significantly shorter than that in the control group (169.3 ± 7.8 min *vs* 180.2 ± 7.5 min, *P* < 0.001). Moreover, the experimental group demonstrated a significantly lower average estimated blood loss than the control group (28.1 ± 8.2 ml *vs* 37.7 ± 8.7 ml, *P* < 0.001). Additionally, the experimental group exhibited a reduced mean postoperative hospitalization compared to the control group (11.2 ± 1.4 d *vs* 12.4 ± 1.3 d, *P* = 0.001). There was a statistically significant difference in the postoperative visual incision length between the two groups, with the experimental group having a smaller median visual incision length than the control group (5.5 cm *vs* 11.0 cm, *P* < 0.001). Median VAS scores were lower in the experimental group on the first postoperative day (3.0 *vs* 4.0, *P* = 0.015). There were no statistically significant differences between the two groups regarding the maximum tumor diameter and time to first flatus after surgery (*P* > 0.05). No positive margins at the distant or circumferential resection margins were found in either group. None of the patients required reoperation.

**Table 3:** Intraoperative and postoperative relevant indicators after PSM.

Variable	Experimental group (n=34)	Control group (n=34)	*P*
Operation time (min)	169.3 ± 7.8	180.2 ± 7.5	<0.001
Estimated blood loss (ml)	28.1 ± 8.2	37.7 ± 8.7	<0.001
Maximum tumor diameter (cm)	6.0 (5.0-7.5)	6.0 (5.0-7.0)	0.862
Postoperative visual incision length (cm)	5.5 (4.5-6.5)	11.0 (9.0-13.0)	<0.001
Positive DRM	0	0	N/A
Positive CRM	0	0	N/A
Time to first flatus (d)	2.0 (1.0-4.0)	2.0 (1.0-4.0)	0.119
VAS score	3.0 (1.0-6.0)	4.0 (2.0-6.0)	0.015
Postoperative hospitalization (d)	11.2 ± 1.4	12.4 ± 1.3	0.001
Reoperation	0	0	N/A

DRM, Distal resection margin; CRM, Circumferential radial margin; VAS, Visual analogue scale; N/A, Not applicable.

### 3.3 Stoma-related indicators after PSM

**Figure 4:**
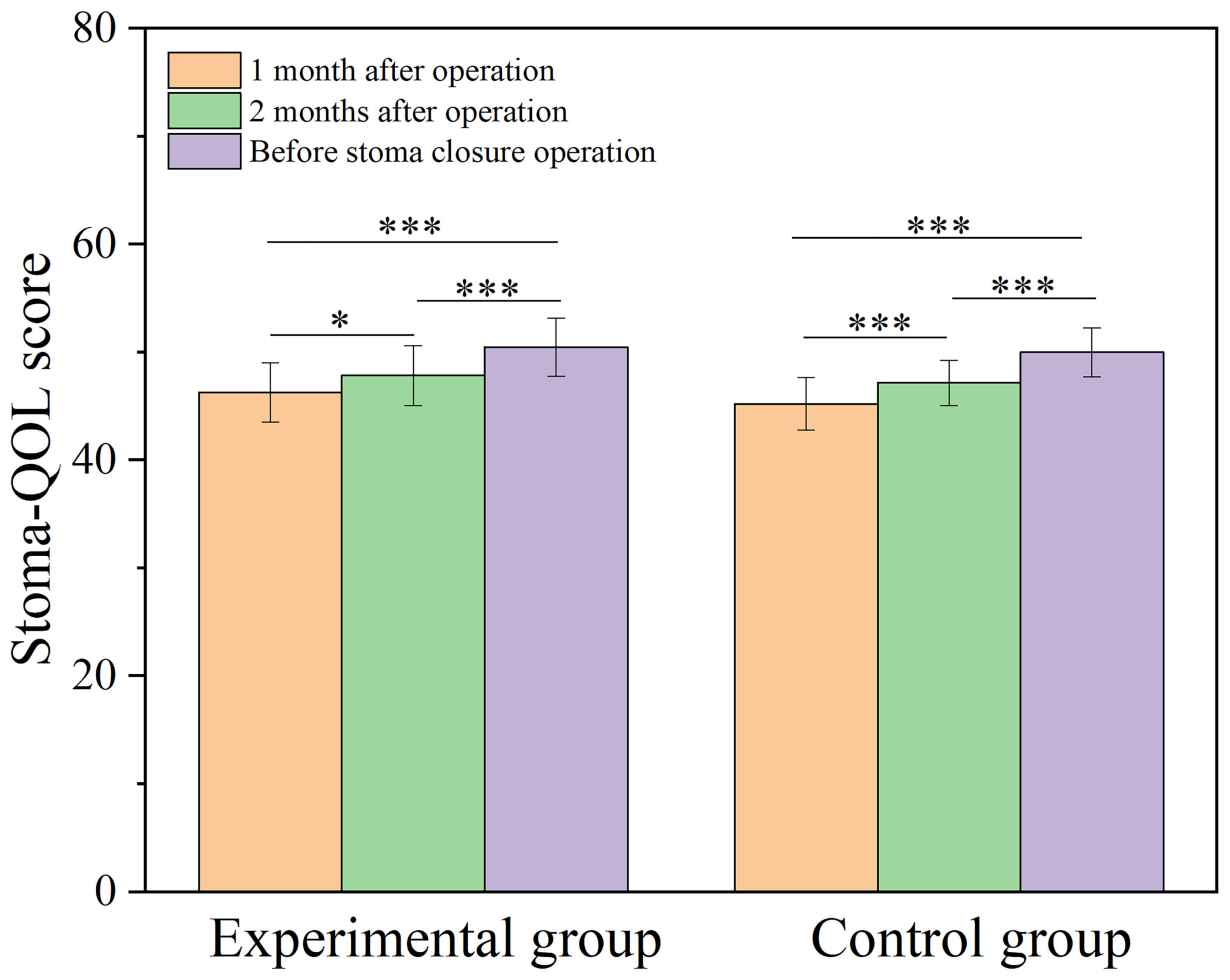
Bar graph comparing Stoma-QOL score at one month, two months postoperatively and before stoma closure operation for both groups. Stoma-QOL: Stoma quality of life; *, *P* < 0.05; ***, *P* < 0.001.

The incidence of stoma complications (stoma retraction, stoma stenosis, stoma infection, stoma necrosis, stoma edema, skin inflammation around the stoma, and parastomal hernia) in both patient groups showed no statistically significant difference (*P* > 0.05). The difference in median DET score between the experimental and control groups was not statistically significant (*P* > 0.05). At postoperative one month and two months, as well as before the stoma closure operation, there were no statistically significant differences in Stoma-QOL scores between the two groups of patients (*P* > 0.05). However, within each group of patients, the Stoma-QOL scores before stoma creation were significantly higher compared to the scores at one month and two months after the operation (*P* < 0.05) (**Figure 4**). Detailed results of the stoma-related indicators for both groups after PSM are presented in **Table 4**.

**Table 4:** Stoma-related indicators after PSM.

Variable	Experimental group (n=34)	Control group (n=34)	*P*
Stoma retraction	0	1 (2.9%)	1.000
Stoma stenosis	0	1 (2.9%)	1.000
Stoma infection	1 (2.9%)	1 (2.9%)	1.000
Stoma necrosis	0	0	N/A
Stoma edema	3 (8.8%)	6 (17.6%)	0.474
Skin inflammation around the stoma	6 (17.6%)	4 (11.8%)	0.493
Parastomal hernia	3 (8.8%)	1 (2.9%)	0.606
DET score	2.0 (1.0-4.0)	2.0 (1.0-4.0)	0.979
Stoma-QOL score			
1 month after operation	46.3 ± 2.7	45.2 ± 2.4	0.096
2 months after operation	47.8 ± 2.8	47.2 ± 2.1	0.260
Before stoma closure operation	50.5 ± 2.7	50.0 ± 2.2	0.409

DET, Discoloration, erosion, and tissue overgrowth; Stoma-QOL, Stoma quality of life; N/A, Not applicable.

## 4 Discussion

Two different incisions were selected for the conventional ostomy surgery and the additional incision for specimen extraction. The main reasons for this choice can be summarized as follows: firstly, the ability to select a more operationally convenient site for specimen extraction; secondly, the difficulty in extracting specimens through the stoma incision when the tumor diameter is substantial, or the mesentery is too thick; and thirdly, the provision of comprehensive protection for both the stoma and abdominal incisions. With the development of laparoscopic techniques and surgical instruments, two methods have emerged for specimen extraction: NOSES and *via* a stoma site (10, 12). However, compared to specimen extraction through the stoma site, NOSES requires a higher learning cost. Previous studies have demonstrated the safety and feasibility of prophylactic ileostomy for specimen extraction in laparoscopic rectal cancer surgery (16–18). However, Wanglin Li et al. reported an increased incidence of peristomal hernia when specimens were extracted through the ileostomy site (19). Specimens extraction *via* the temporary ileostomy site were safety in the study by Kil-Yong Lee et al. (17). However, it increased the incidence of stoma complications without significant difference compared to retrieval through an alternative incision. Chao Liu et al. also reported that prophylactic ileostomy for specimen extraction saved procedure time and reduced intraoperative bleeding and proposed that a lower midline incision and left lower abdominal incision were ideal for ileostomy (18). In cases where tumors had a huge diameter or when it was challenging to extract the intestinal tube and mesentery through a conventional-sized ostomy, this study aimed to explore the safety and feasibility of specimen extraction *via* a U-Plus skin bridge loop ileostomy after laparoscopic LAR, comparing it with specimen extraction through an additional abdominal incision.

Internationally, the Pfannestiel incision is frequently employed for specimen extraction, with pertinent research advocating it as the method of choice for minimally invasive colorectal cancer surgeries (20). Traditionally, our institution has predominantly utilized a midline incision in the lower abdomen for specimen extraction. However, we have expanded our approach to include standard stoma, U-Plus stoma, Pfannestiel incision, and natural orifice techniques in recent years. Due to the relatively few cases in our institution where the Pfannestiel incision was employed for specimen extraction, this study was designed to enhance comparative analysis by selecting cases where specimen extraction was performed *via* the midline lower abdominal incision and the U-Plus skin bridge loop ileostomy. 

The skin bridge loop colostomy was first reported by Milner et al. in 2006 (21). In 2014, Pace et al. described the surgical details of the skin bridge loop ileostomy (7). Subsequent studies have further confirmed the safety and feasibility of the skin bridge loop ileostomy and its ability to reduce postoperative complications associated with the stoma (8, 9). In our study, all patients underwent preoperative stoma site marking. A systematic review and meta-analysis reported that preoperative stoma site marking could reduce the overall complication rate by 53% and skin-related issues by 59% (22).

In this study, 44 patients with rectal cancer underwent successful specimen extraction *via* the U-Plus skin loop ileostomy after laparoscopic LAR (experimental group). The experimental group exhibited shorter operation time and lower estimated intraoperative blood loss than the control group. This can be attributed to the absence of an additional abdominal incision for specimen extraction, eliminating the need for incision and suturing procedures, thereby reducing surgical time. Furthermore, concerns regarding vascular damage and other blood loss during the creation of an additional incision were avoided. The postoperative hospitalization duration of the control group is longer than that of the experimental group, possibly because patients in the control group have more postoperative wounds, slower recovery, which extends the postoperative hospitalization duration. Additionally, the postoperative visible incision length was shorter in the experimental group compared to the control group, aligning with modern minimally invasive principles and enhancing cosmetic outcomes.

A parastomal hernia is an incisional hernia associated with an abdominal wall stoma and is one of the common complications following stoma surgery. The occurrence rate of parastomal hernia ranges from 0% to 48.1%, primarily depending on the type of stoma and the duration of follow-up (23). Some parastomal hernias may not present with relevant signs or clinical symptoms and require CT scans for detection. These hernias do not necessarily require exceptional management. According to the research conducted by Li et al., factors such as stoma specimen extraction site, BMI, and blood transfusion are considered risk factors for the occurrence of parastomal hernia (19). While the findings of this study indicate a marginally elevated incidence of parastomal hernia in the experimental group relative to the control group, the disparity does not achieve statistical significance. This could be attributed to the enlargement of the stoma, leading to an increased occurrence of parastomal hernia. Stoma closure procedures are generally performed 3-6 months after stoma formation, and the issues caused by parastomal hernia do not contribute to increased reoperation among patients. These issues are temporary and can be addressed during the stoma closure procedure.

In this study, we innovatively employed the U-Plus skin bridge loop ileostomy, which involves creating an additional incision on the upper side of the U-shaped incision of the skin bridge loop ileostomy, extending upward along the midline. This allows for the selection of an appropriate length based on tumor size and intestinal diameter, thus avoiding specimen compression due to a small stoma. We also utilized an incision protector to ensure adherence to sterile and tumor-free principles. The reason for using a longitudinal incision is that a transverse incision would sever the rectus abdominis muscle and other related structures, compromising the integrity of the abdominal wall and potentially increasing the occurrence rate of parastomal hernia postoperatively.

No significant differences were observed in the Stoma-QOL score between the two groups at one month and two months postoperatively, as well as prior to the stoma closure surgery. This indicates that the expansion of the ileostomy did not have a noticeable impact on the patient’s quality of life. Furthermore, over time, there was an increase in the Stoma-QOL score compared to before (**Figure 4**), which could be attributed to the fact that all patients underwent prophylactic ileostomy. As the time for stoma closure approached, the patients’ psychological issues improved more noticeably.

Nevertheless, this is a single-center retrospective study, making it challenging to avoid retrospective bias. We collected all dates clinically and were not pre-designed. Future research endeavors must encompass prospective and randomized multicenter studies to fortify the integrity of our findings. It is a novel approach to extracting specimens for patients with large tumors or thickened mesentery in rectal cancer.

## 5 Conclusion

In summary, using the U-Plus skin bridge loop ileostomy technique after laparoscopic LAR for rectal cancer is a safe and viable approach. This method shortens the operative time, reduces blood loss, and decreases postoperative visual incision length, improving cosmetic outcomes. It is a novel approach to extracting specimens for patients with large tumors or thickened mesentery in rectal cancer.

## Data availability statement

The original contributions presented in the study are included in the article/Supplementary Material. Further inquiries can be directed to the corresponding author.

## Ethics statement

The studies involving humans were approved by The Ethics Review Board of Jingzhou Hospital Affiliated to Yangtze University. The studies were conducted in accordance with the local legislation and institutional requirements. The participants provided their written informed consent to participate in this study. Written informed consent was obtained from the individual(s) for the publication of any potentially identifiable images or data included in this article.

## Author contributions

SX: Software, Writing – review & editing, Conceptualization, Data curation, Formal Analysis, Investigation, Methodology, Visualization, Writing – original draft. SH: Writing – original draft, Investigation, Methodology, Conceptualization, Data curation, Formal Analysis. HY: Conceptualization, Methodology, Validation, Writing – review & editing, Project administration. WL: Data curation, Investigation, Writing – review & editing. XZ: Data curation, Investigation, Writing – review & editing.
